# *PAX6* missense variants in two families with isolated foveal hypoplasia and nystagmus: evidence of paternal postzygotic mosaicism

**DOI:** 10.1038/s41431-020-00737-1

**Published:** 2020-10-06

**Authors:** Dulce Lima Cunha, Nicholas Owen, Vijay Tailor, Marta Corton, Maria Theodorou, Mariya Moosajee

**Affiliations:** 1 UCL Institute of Ophthalmology, London, UK; 2 Moorfields Eye Hospital NHS Foundation Trust, London, UK; 3 UCL Experimental Psychology, London, UK; 4 Department of Genetics & Genomics, Instituto de Investigación Sanitaria-Fundación Jiménez Díaz University Hospital – Universidad Autónoma de Madrid (IIS-FJD, UAM), Madrid, Spain; 5 Centre for Biomedical Network Research on Rare Diseases (CIBERER), Madrid, Spain; 6 Great Ormond Street Hospital for Children NHS Foundation Trust, London, UK

**Keywords:** Mutation, Genetic testing, Mutation, Genetic testing, Mutation

## Abstract

PAX6 is considered the master regulator of eye development, the majority of variants affecting this gene cause the pan-ocular developmental eye disorder aniridia. Although no genotype-phenotype correlations are clearly established, missense variants affecting the DNA-binding paired domain of PAX6 are usually associated with non-aniridia phenotypes like microphthalmia, coloboma or isolated foveal hypoplasia. In this study, we report two missense heterozygous variants in the paired domain of PAX6 resulting in isolated foveal hypoplasia with nystagmus in two independent families: c.112 C > G; p.(Arg38Gly) and c.214 G > C; p.(Gly72Arg) in exons 5 and 6, respectively. Furthermore, we provide evidence that paternal postzygotic mosaicism is the cause of inheritance, with clinically unaffected fathers and reduced affected allele fraction. This work contributes to increase the phenotypic spectrum caused by *PAX6* variants, and to our knowledge, is the first report to describe the presence of postzygotic parental mosaicism causing isolated foveal hypoplasia with nystagmus. These results support the growing evidence that suggest an overestimation of sporadic cases with *PAX6* variants, which has strong implications for both genetic counselling and family planning.

## Introduction

PAX6 is a member of the highly conserved paired-box (PAX) family of transcription factors and is critical for neural and ocular development [1]. The *PAX6* gene (OMIM 607108) is composed of 14 exons that encode a protein containing two DNA-binding domains, the paired domain (PD) and the homeodomain, followed by a proline-serine-threonine rich domain with transactivation properties [2]. Variants in *PAX6* have been reported across the coding sequence of the gene, the majority being loss of function [3, 4]. *PAX6* haploinsufficiency tends to cause aniridia (OMIM 106210), a pan-ocular disorder characterised by partial or complete iris hypoplasia, foveal hypoplasia and nystagmus, with subsequent development of cataracts, glaucoma and corneal keratopathy [5, 6]. In contrast, missense variants in *PAX6* are mostly concentrated in exons 5 and 6, which encode for the PD, and are largely associated with non-aniridia phenotypes such as microphthalmia, ocular coloboma (OMIM 120200), foveal hypoplasia with or without anterior segment anomalies and/or cataract (OMIM 136520) [3, 7]. *PAX6* variants are associated with high phenotypic variability, complicating the establishment of genotype-phenotype correlations and hindering clinical diagnosis and management [6].

In autosomal dominant disorders, it is estimated that parental mosaicism can be found in up to 17% of sporadic cases with apparent de novo variants [8]. In patients with *PAX6*-related aniridia, two-thirds are familial cases, while the remaining are sporadic comprising of de novo point variants or, less frequently, larger deletions encompassing *PAX6* and the neighbouring gene *WT1*, resulting in Wilms tumour, aniridia, genitourinary anomalies and mental retardation (WAGR) syndrome [5, 9]. The presence of parental mosaicism in three *PAX6*-affected individuals (two with aniridia and one with microphthalmia) was recently reported by Tarilonte et al., where affected allele fractions in unaffected or mildly affected fathers ranged from 10 to 30% in different tissues analysed [10]. In this study, we report two independent families presenting with isolated foveal hypoplasia and nystagmus caused by missense heterozygous changes in *PAX6* predicted to affect the PD. Furthermore, we show that the presence of paternal postzygotic mosaicism is the cause of inheritance.

## Material and methods

### Patient description

This study was approved by Moorfields Eye Hospital and the National Research Ethics Committee and was conducted in adherence to the tenets of the Declaration of Helsinki; informed written consent was obtained from all participants. Ophthalmic evaluation included full orthoptic assessment, refraction, best corrected visual acuity using LogMAR, slit lamp examination, and fundus examination recorded with anterior segment and fundus colour imaging. Investigations included eye movement recordings (EMR), electrophysiology, spectral domain optical coherence tomography (SD-OCT) and fundus autofluorescence.

### Molecular screening

Sanger sequencing of the probands DNA was performed to screen the *PAX6* gene at the NHS Wessex Regional Genetics Laboratory (WRGL). *PAX6* transcript NM_000280.4/ ENST00000643871.1 was used for variant nomenclature and exon numbering. The gnomAD data set (https://gnomad.broadinstitute.org/; accessed Nov 2019) was used to estimate the prevalence of the identified variants. Deleteriousness was scored using in silico algorithms SIFT, PolyPhen2, MutationTaster, PROVEAN and CADD scoring [11]. Variants were submitted to ClinVar with accession numbers VCV000800413.1 and VCV000637045.2. Siblings and parents had familial testing to confirm segregation. Urine, buccal swab and hair follicles were further collected from the fathers of both probands and genomic DNA extracted with QiAmp DNA MicroKit (Qiagen, Germany), amplified by PCR using standard conditions and variants were confirmed by direct sequencing. Droplet digital PCR (ddPCR) analysis was performed in DNA from blood obtained from Family 1 father (1–2) and unaffected sibling (1–4) using a custom-designed TaqMan SNP Genotyping Assay (Applied Biosystems, CA, USA) for the variant NM_000280.4:c.214 G > C as previously described [10].

## Results

### Clinical findings

**Fig. 1:**
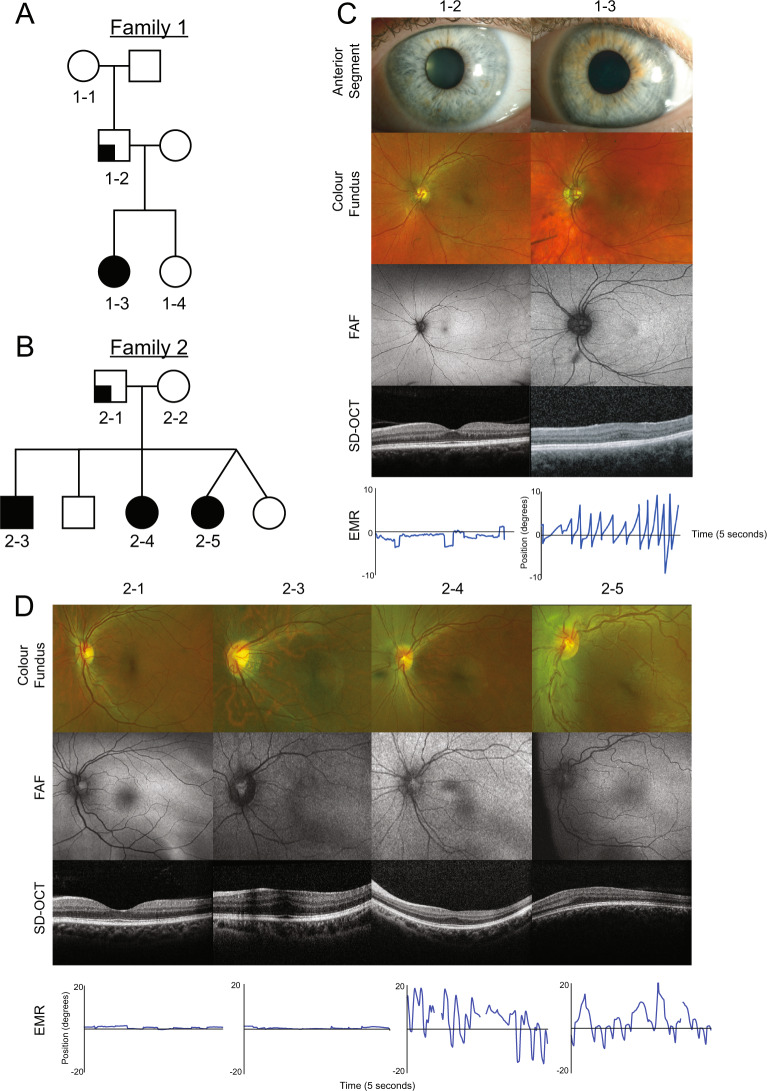
Pedigree and clinical imaging of probands and members of Families 1 and 2 displaying the left eye only (although there was symmetry between both eyes). **a** Three-generation Caucasian British non-consanguineous pedigree, with one affected patient (proband 1–3). **b** Two-generation Somalian non-consanguineous pedigree with three affected siblings (proband 2–4). **c** Family 1, father (1–2) and proband (1–3) showing anterior segment photos with normal iris architecture, colour fundus image, fundus autofluorescence (FAF), spectral domain optical coherence tomography (SD-OCT) and eye movement recordings (EMR). **d** Family 2 with father (2–1), eldest brother (2–3), proband (2–4) and younger sister (2–5) showing colour fundus, FAF, SD-OCT and EMR.

Two unrelated families were identified (Fig. 1a, b), where the probands (1–3 and 2–4) exhibited nystagmus and reduced vision from infancy. Family 1 were of British Caucasian descent, and Family 2 were Somalian, neither were consanguineous. Detailed ophthalmological features of probands and family members are presented in Table 1 and Fig. 1. The best corrected visual acuity ranged between 0.00 and 1.00 LogMAR in molecularly confirmed affected members. There was significant intra-familial phenotypic variability with patient 2–3 from Family 2 being more mildly affected than his siblings. All affected patients had a refractive error varying between mild-moderate hypermetropic to myopic astigmatism. SD-OCT of affected patients revealed variable degrees of foveal hypoplasia, graded according to Thomas et al. [8]. None had any iris abnormalities, anterior segment dysgenesis or signs of cataract. No systemic abnormalities were present.

**Table 1:** Clinical findings of probands and members of Family 1 and 2.

	Age	Gender	BCVA OD	BCVA OS	Refraction	Colour OD	Colour OS	Strabismus	Nystagmus	IOP OD	IOP OS	Foveal Hypoplasia	EDT
Family 1													
1-2	53	Male	0.00	0.00	NA	17/17	17/17	No deviation	Square wave jerks	NA	NA	Normal	Not undertaken
1-3 (Proband)	24	Female	1.00	0.70	Myopic astigmatism	16/17	16/17	Right exotropia	Jerk with extended foveation	18	20	Grade 3	No evidence of crossed asymmetry and no generalised rod dysfunction
Family 2													
2-1	44	Male	0.00	0.00	NA	17/17	17/17	No deviation	None	NA	NA	Normal	Not undertaken
2-3	22	Male	0.00	0.20	Myopia	17/17	17/17	No deviation	Intermittent jerk	9	14	Grade 1	No evidence of crossed asymmetry and no generalised rod dysfunction
2-4 (Proband)	20	Female	0.60	0.60	R: ∞/−1.75 × 5 L: −2.00/−2.00 × 180	12/17	8/17	Right exotropia	Pendular asymmetric	21	19	Grade 1	No evidence of crossed asymmetry and no generalised rod dysfunction
2-5	15	Female	0.20	0.54	R: + 3.25/−1.25 × 180 L: + 0.75DS	NA	NA	Left exotropia	Jerk with extended foveation	14	18	Grade 2	NA

*BCVA* best corrected visual acuity given in LogMAR, *OD* right eye, *OS* left eye, *OU* both eyes open, *Colour* colour vision measured with Ishihara Chart, *IOP* intraocular pressure, *EDT* electrodiagnostic test including electroretinogram (ERG) and visual evoked potentials (VEP), *NA* not available.

### Genetic analysis

**Fig. 2:**
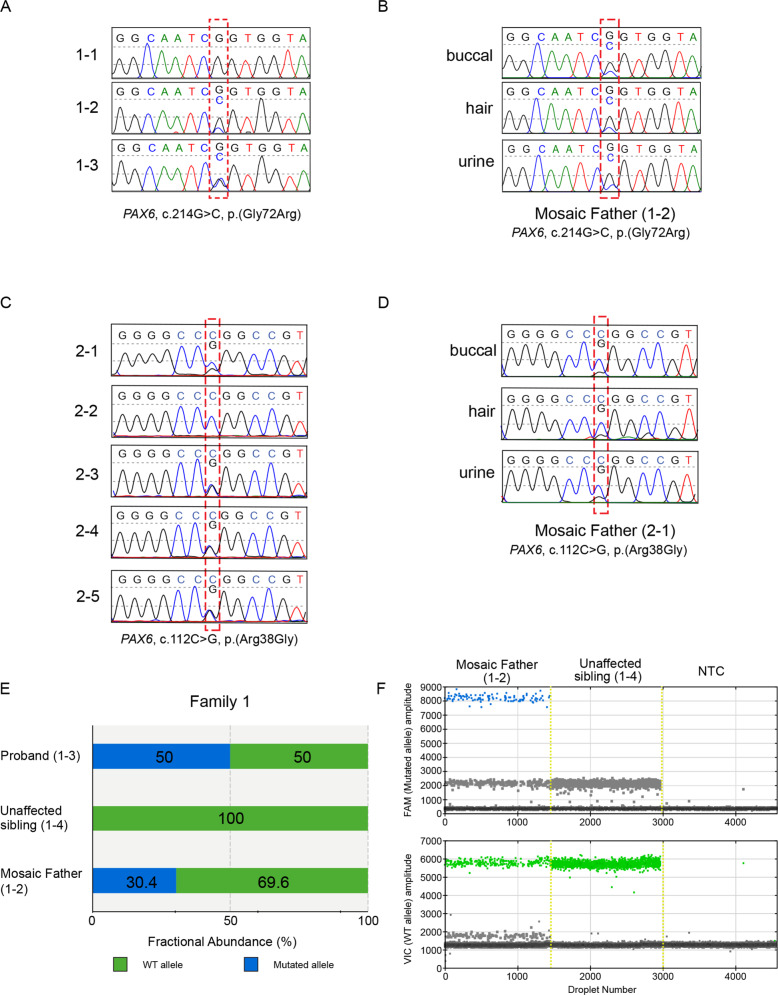
Genotype information of probands and members of Families 1 and 2. **a** DNA chromatograms from blood of all the tested members of Family 1, the position of the variant (c.214 G > C) in exon 6 of *PAX6* bounded by the red dotted box. **b** DNA chromatograms from the father (1–2) of the proband from Family 1 from somatic tissues: buccal, hair and urine epithelia. **c** DNA chromatograms from blood samples from tested members of Family 2 highlighting *PAX6* c.112 C > G variant within the red dotted box. **d** DNA chromatograms from the father (2–1) of the proband from Family 2 from somatic tissues: buccal, hair and urine epithelia. **e** Quantification of allele abundance by Digital Droplet PCR (ddPCR) for Family 1. Allele percentages of suspected mosaic father (1–2), non-carrier sibling (1–4) and proband (1–3). **f** Fluorescence amplitude plot showing presence of FAM-labelled droplets in blue (affected allele) in mosaic father (1–2) and absent in non-carrier sibling (1-4, wildtype) as well as the negative control (no template control, NTC). VIC-labelled droplets (green), containing the wildtype allele, were detected in both individuals. Unlabelled droplets are represented in grey.

Genotyping of proband 1–3 from Family 1 revealed a heterozygous missense variant in exon 6 of *PAX6*, NM_000280.4:c.214 G > C; p.(Gly72Arg) (Fig. 2a). DNA analysis of proband 2–4 from Family 2 showed a heterozygous missense variant in exon 5 of *PAX6*, NM_000280.4:c.112 C > G; p.(Arg38Gly) (Fig. 2c). Both variants are predicted pathogenic (Supplementary Table 1); the probands from each family were included in a recent report of genotypes solved by the WRGL [12]. Neither of the probands’ parents presented with any ocular abnormalities or visual dysfunction; the mothers were homozygous for the normal allele. However, a second low peak with the respective affected allele was visible in the sequences of both fathers: G > C in 1–2 (Family 1) and C > G in 2–1 (Family 2), pointing to postzygotic mosaicism in these individuals (Fig. 2a, c).

### Evidence of parental mosaicism

To further investigate the presence of mosaicism in individuals 1–2 and 2-1 we analysed DNA extracted from urine epithelial cells, hair follicles and buccal epithelial cells. Sanger sequencing confirmed the presence of a smaller peak corresponding to the affected allele in all samples tested (Fig. 2b, d). ddPCR was then performed to quantify the fraction of the affected allele using DNA from the blood of individual 1–2 (Family 1). ddPCR could not be performed for individual 2-1 (Family 2), due to repeated QC failure during synthesis of the Taqman assay for the variant NM_000280.4:c.112 C > G. Analysis of individual 1–2 confirmed the presence of mosaicism, showing an affected allele fraction of 30.4% (Fig. 2e). DNA from the proband’s unaffected sister (1–4) was used as a control and shows no detection of affected allele (Fig. 2f).

## Discussion

We report two families with isolated foveal hypoplasia and nystagmus (OMIM 136520) caused by two different *PAX6* missense variants predicted to affect the PD [12]. Reported variants affecting the amino acid Gly72 are largely associated with nystagmus and foveal hypoplasia (c.214 G > A, p.(Gly72Ser) and c.214 G > T, p.(Gly72Cys)) [12, 13], and combined with high myopia (c.215 G > T, p.(Gly72Val)) [14]. For the amino acid Arg38, variants have been associated with nystagmus and congenital cataracts, or bilateral microphthalmia (c.113 G > A, p.(Arg38Gln)) [15, 16]; c.112 C > T, p.(Arg38Trp) has been identified in patients with nystagmus, foveal hypoplasia, microcornea and optic nerve hypoplasia [17, 18] or iris coloboma with cataracts [16]. Our findings support the evidence that PD missense variants, particularly in the C-terminal subdomain of the PD, are more frequently associated with non-aniridia phenotypes [3, 4]. These variable phenotypes are likely due to the physicochemical differences of the resulting amino acid and its effect in the DNA-binding activity of the PD [19]. However, it has recently been noted that some missense changes are likely to affect splicing mechanisms, which could in fact result in loss of function variants, perhaps explaining the cases presenting with iris phenotype [20].

Germline or postzygotic mosaicism has long been suspected to be a cause for apparent *de novo* cases with *PAX6* variants [21, 22], but only recently it was confirmed by analysis of different somatic tissues of suspected carriers [10]. We report the presence of postzygotic mosaicism in two clinically unaffected individuals, with father 1–2 carrying an estimated affected allele fraction of 30.4%, which combined with the presence in different tissues, points to an early postzygotic event. EMR detected square wave jerks at a frequency higher in the otherwise unaffected father 1–2 compared to normal unaffected individuals, which could suggest the affected allele is also present in his cells of ocular origin, resulting in a mild defect. Since *PAX6* is a dose-sensitive gene, it could be hypothesised that if enough protein could be restored to a level equivalent to 70% wildtype allele, this could be enough to alleviate clinical disease. This may be possible through strategies such as nonsense suppression or gene augmentation.

To our knowledge, this is the first report of paternal mosaicism involving *PAX6* variants causing isolated foveal hypoplasia with nystagmus. This work further supports the underestimation of mosaicism rates in *PAX6* sporadic cases, which has important implications for genetic counselling, family planning and disease management. Until the genetic diagnosis is confirmed in patients, sporadic cases must be investigated for WAGR syndrome with serial renal ultrasound monitoring of potential Wilms tumour. The presence of paternal mosaicism highlights the importance of performing genetic screening of healthy parents of sporadic cases, since there could be a higher recurrence risk in future offspring.

## Supplementary information

Supplementary Table 1
